# Surveillance of Fontan-associated liver disease: current standards and a proposal from the European Society of Paediatric Radiology Abdominal Task Force

**DOI:** 10.1007/s00247-021-05173-x

**Published:** 2021-10-15

**Authors:** Giulia Perucca, Charlotte de Lange, Stéphanie Franchi-Abella, Marcello Napolitano, Michael Riccabona, Damjana Ključevšek, Seema Toso, Jochen Herrmann, Samuel Stafrace, Kassa Darge, Maria Beatrice Damasio, Costanza Bruno, Magdalena Maria Woźniak, Luisa Lobo, Donald Ibe, Anne M. Smets, Philippe Petit, Lil-Sofie Ording Müller

**Affiliations:** 1 Department of Pediatric Radiology, Regina Margherita Children’s Hospital, Turin, Italy; 2 Department of Radiology and Clinical Physiology, Queen Silvia Children’s Hospital, Sahlgrenska University Hospital, Göteborg, Sweden; 3 Pediatric Radiology Department, Hôpital Bicêtre, Hôpitaux Universitaire Paris-Sud, Assistance Publique Hôpitaux de Paris, Le Kremlin-Bicêtre, France; 4 Department of Paediatric Radiology and Neuroradiology, V. Buzzi Children’s Hospital, Milan, Italy; 5 Department of Radiology, Division of Pediatric Radiology, Medical University Graz and University Hospital LKH, Graz, Austria; 6 Department of Radiology, University Children’s Hospital Ljubljana, Ljubljana, Slovenia; 7 Department of Pediatric Radiology, University Hospital of Geneva, Geneva, Switzerland; 8 Department of Pediatric Radiology, University Hospital Hamburg Eppendorf, Hamburg, Germany; 9 Department of Diagnostic Imaging, Sidra Medicine, Doha, Qatar; 10 Weill Cornell Medicine, Doha, Qatar; 11 Department of Radiology, The Children’s Hospital of Philadelphia, University of Pennsylvania, Philadelphia, PA USA; 12 Radiology Department, IRCCS Istituto Giannina Gaslini, Genoa, Italy; 13 Department of Radiology, Azienda Ospedaliera Universitaria Integrata Verona (AOUI), Verona, Italy; 14 Department of Pediatric Radiology, Medical University of Lublin, Lublin, Poland; 15 Serviço de Imagiologia Geral, Hospital de Santa Maria–Centro Hospitalar Universitário Lisboa, Norte (CHULN), Lisbon, Portugal; 16 Department of Radiology, Silhouette Diagnostic Consultants, Abuja, Nigeria; 17 Department of Radiology and Nuclear Medicine, Amsterdam UMC, University of Amsterdam, Amsterdam, the Netherlands; 18 Aix Marseille Université, AP-HM, Equipe d’Accueil 3279 - IFR 125, Hôpital Timone Enfants, Service d’Imagerie Pédiatrique et Prénatale, Marseille, France; 19 Unit for Paediatric Radiology, Department of Radiology, Oslo University Hospital, Rikshospitalet, PB 4950 Nydalen, 0424 Oslo, Norway

**Keywords:** Adolescents, Children, Cirrhosis, Fontan procedure, Hepatocellular carcinoma, Liver, Liver fibrosis, Magnetic resonance imaging, Ultrasound

## Abstract

Since Francis Fontan first introduced the eponymous technique, the Fontan procedure, this type of surgical palliation has allowed thousands of children affected by specific heart malformations to reach adulthood. Nevertheless, abdominal, thoracic, lymphatic and neurologic complications are the price that is paid by these patients. Our review focuses on Fontan-associated liver disease; the purpose is to summarize the current understanding of its physiopathology, the aim of follow-up and the specific radiologic follow-up performed in Europe. Finally, we as members of the Abdominal Task Force of the European Society of Paediatric Radiology propose a consensus-based imaging follow-up algorithm.

## Introduction

**Fig. 1:**
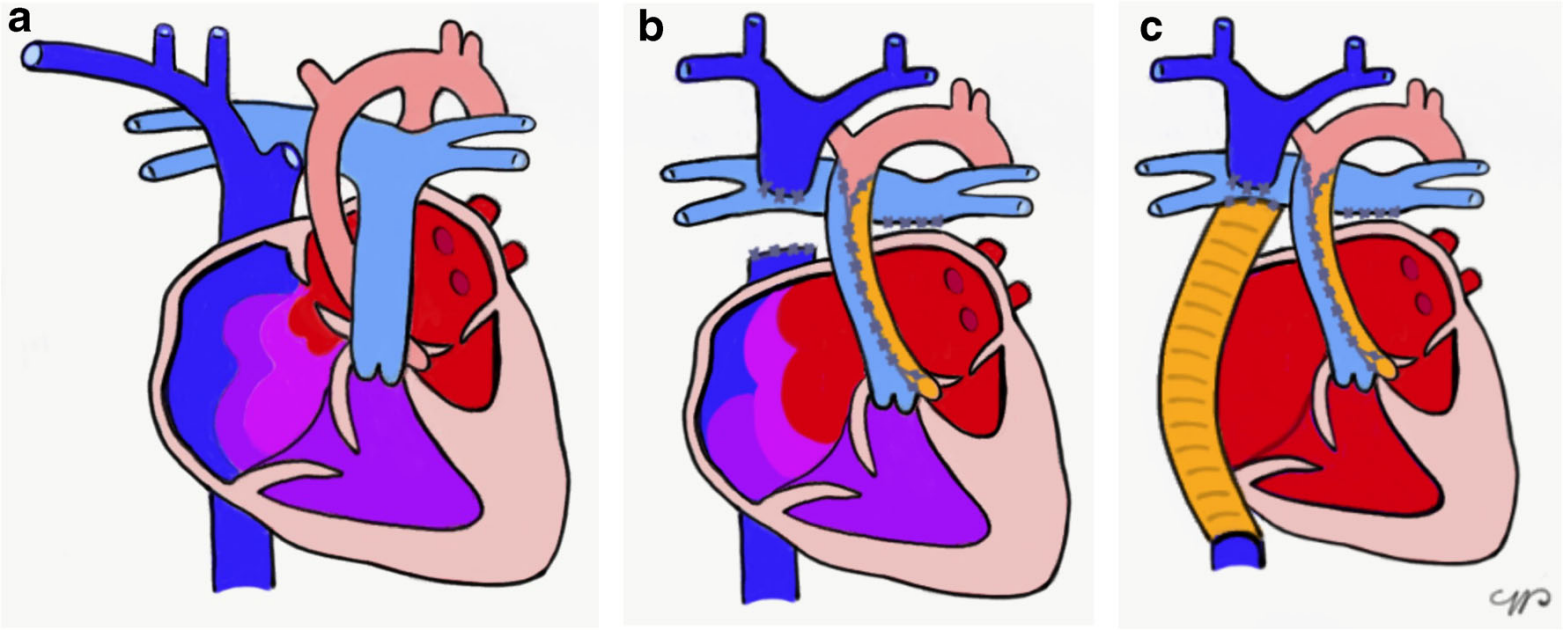
Fontan surgery, simplified. **a–c** In hypoplastic left heart syndrome (**a**) the total cavo-pulmonary connection is reached through enlargement of the atrial septal defect (**b**), reconstruction of the aorta with a homograft patch, and connection of the superior and inferior venae cavae — the latter through a conduit (**c**) to the pulmonary artery

The Fontan procedure was initially performed in 1968 for children affected by tricuspid atresia [1]. Since then, the surgical technique has been modified [2] and currently consists of a series of planned surgical interventions that occur from shortly after birth until 2–4 years of age, resulting in a direct connection between the caval veins and the pulmonary arteries (Fig. 1). This represents a palliation for children affected by pathologies with a single functional ventricle, the most common being hypoplastic left heart syndrome. The only definitive treatment is heart transplantation.

Venous congestion caused by this new circulation, as well as the pre-, peri- and postoperative cardiac conditions, can cause hepatic fibrosis, often leading to the development of liver cirrhosis. The aim of liver imaging in Fontan patients is to assess the presence and progression of fibrosis, with close surveillance for hepatic nodules to detect potential malignancy.

Although the Fontan procedure is now more than 50 years old, a universally accepted follow-up imaging protocol of the liver has not been established. Although some proposed follow-up algorithms have been published recently in North America [3, 4], these guidelines do not reflect European practice, as highlighted by a recent European Society of Paediatric Radiology (ESPR) Abdominal Task Force survey [5].

A common and more uniform liver imaging follow-up protocol would allow these children to have a more homogeneous diagnosis and ultimately more harmonized treatment across Europe. This would, in addition, improve and increase our knowledge of this pathology and permit clinicians to adjust liver surveillance protocols based on more solid and comparable data.

The main consequence of the artificially created circulation is an increased systemic venous pressure and a decreased systemic arterial output [6]. In addition, the pre-, peri- and post-surgical abnormal hemodynamic condition is likely to contribute to the complications [7]. These can affect several organs [8], though the focus of this paper is on Fontan-associated liver disease.

The effect on the liver, from the increased non-pulsatile pressure in the superior and inferior venae cavae caused by direct communication with the pulmonary arterial system, is similar to what is observed in any cause of congestive hepatopathy on imaging. The typical mottled, nutmeg-pattern of liver parenchyma on contrast-enhanced CT or MRI is a result of the increased pressure within the central hepatic veins, which causes sinusoidal congestion. This results in a decreased venous portal inflow, increased compensatory arterial hepatic inflow and a decreased venous hepatic outflow [9]. Sinusoidal congestion and dilatation precede the appearance of fibrosis. Fibrosis develops initially around central hepatic venules, then extends to the entire lobule through bridging fibrosis. Portal fibrosis is also observed in Fontan patients and is thought to be related to the portal hypertension resulting from chronic systemic venous hypertension [10]. Ultimately, cirrhosis develops as a result of regenerative tissue and bridging fibrosis. Venous thrombosis has also been reported. However, it is still unclear whether this is an additional cause of fibrosis onset rather than a consequence [11, 12]. In Fontan patients, the chronic tissue hypoxia caused by low cardiac output results in hepatocyte injury and contributes to the development of fibrosis and finally, in some cases, cirrhosis.

It is important to bear in mind that the distribution of those changes throughout the liver is heterogeneous, so that within the same organ one might find alternate areas of normal liver, fibrosis and cirrhosis. Hence, liver biopsy results might not be representative of true liver disease.

The timing of appearance of liver changes is unknown and varies among individuals, influenced both by the pre-surgical status and the surgical outcome. It is possible to see signs of liver fibrosis early, even 5 years after surgery [13]. Nearly all Fontan patients develop liver complications and these tend to be more severe with time, but they usually do not correlate with symptoms [14].

Cirrhosis is the prerequisite for developing hepatocellular carcinoma (HCC), with an incidence of 1–5% per year in Fontan patients [15, 16]. The altered hepatic vasculature, on the other hand, favors the appearance of hypervascular nodules, considered to be regenerative nodules and focal (nodular) hyperplasia-like nodules [17]. Because of the liver congestion, diagnosis on imaging can be challenging — hyperenhancing nodules on contrast-enhanced CT and MRI can demonstrate washout on portal venous or delayed phase images regardless of their benign or malignant nature [18, 19]. It is important to differentiate these from HCC because Fontan patients are at risk of developing HCC at a young age, and mortality following HCC diagnosis is high [16]. A few cases of HCC diagnosed in the second decade of life have been reported, the youngest child being 13 years old [20–23].

## Imaging tools

### Abdominal ultrasound

Abdominal US is the easiest noninvasive imaging screening tool for liver disease. The aim is to look for signs of portal hypertension, diffuse or focal structural liver changes, and liver nodules, although the accuracy of US in the detection of liver nodules is inferior to that of CT and MRI [24, 25].

The choice of transducer is adapted to the size of the child. Both convex and linear transducers are used; high-resolution linear probes help evaluate for the presence of liver nodules, assess the liver surface and contour, and detect portosystemic shunts that are more easily seen.

Abdominal US evaluates liver size and echogenicity, the presence of hepatic nodules, the diameter of the main portal vein and hepatic veins, and the relevant aspects of the biliary tree. The hepatic veins, portal vein and hepatic artery are examined on color and pulsed Doppler to evaluate for portal hypertension. If present, portosystemic shunts and collaterals are depicted [26]. Spleen size is measured and the presence of ascites documented. A systematic evaluation of the whole abdomen is performed, not to miss other significant findings.

*Practical point: liver US is a safe, inexpensive and a reliable tool for detecting signs of fibrosis/cirrhosis and portal hypertension as well as larger liver nodules and can be used as a screening tool.*

### Contrast-enhanced ultrasound

In anyone at high risk for HCC, when a new liver nodule measuring 10 mm or more is detected on gray-scale US, one possible diagnostic tool to establish its benign or malignant nature is contrast-enhanced ultrasound (CEUS) [27]. The US contrast agent SonoVue (sulphur hexafluoride gas microbubbles; Bracco, Milan, Italy) is used off-label in Europe for intravenous applications in children younger than 18 years. In the United States, the same contrast agent (under the name Lumason; Bracco Diagnostics, Monroe Township, NJ) has been approved by the Food and Drug Administration for characterizing focal liver lesions in both children and adults. Written consent might be required for off-label use.

Some considerations need to be made in the specific context of Fontan patients. First, a focal liver lesion is generally suspected to be malignant when it shows heterogeneous contrast enhancement and early washout phase [28]. However, as mentioned, in Fontan patients benign hyperenhancing nodules can be mistaken for malignant lesions because they can also show some contrast washout on post-arterial images [18]. Second, the maximum number of nodules that can be characterized with CEUS is usually two to three, but in many children these nodules are more numerous and some might not be visible on US. Last, according to the European Medicines Agency (EMA), SonoVue should not be used in people with known right-to-left shunts [29]. However, this contraindication has been removed in the United States. Lumason is now labeled only with a warning for the theoretical risk of systemic microvascular obstruction in people with intracardiac shunts [30]. Moreover, intravenous agitated saline is routinely and safely used for detecting intracardiac shunts [31] and the increased risk for systemic embolization has not been proved [32].

*Practical point: Liver CEUS can be used (off-label in Europe) for characterizing suspicious liver nodules in dedicated centers if permissible under local regulations and when major right-to-left shunts have been ruled out. However, it is to be noted that benign nodules in Fontan liver disease might show some post-arterial washout.*

### Magnetic resonance imaging

The basic liver MRI protocol includes T1-weighted sequences in and out of phase, T2-weighted sequences in two planes, balanced steady-state free precession (SSFP) sequences, diffusion-weighted imaging (DWI), and a dynamic acquisition during contrast injection using T1-weighted three-dimensional (3-D) gradient echo sequences with Dixon technique/fat suppression. Children younger than 6 years usually need sedation; the technique must be adapted to the size and age of the child and the smallest possible coil should be used [33].

Regarding MRI contrast agents, gadolinium-based extracellular agents provide similar information to iodinated contrast agents in CT. Macrocyclic agents are preferred to decrease the risk of gadolinium brain deposition, which has been more frequently associated with linear agents [34]. Iterative injection of contrast agent, however, should always be carefully evaluated, especially in children with chronic diseases.

Hepatobiliary contrast agents, such as gadobenate dimeglumine (Gd-BOPTA/Dimeg, MultiHance; Bracco, Milan, Italy) and gadolinium ethoxybenzyl diethylenetriamine pentaacetic acid (Gd-EOB-DTPA, Primovist/Eovist; Bayer Schering Pharma, Berlin, Germany) have a different metabolism because they are partly excreted by the kidneys, and partly by the hepatocytes in the biliary tract. MultiHance has been approved in children older than 2 years, whereas Primovist/Eovist is used off-label in some countries for children younger than 18 years, usually after acquisition of written consent. The hepatic uptake of MultiHance is 2–4% of the injected dose; it is 50% for Primovist/Eovist.

Apart from the vascular enhancement pattern, hepatobiliary contrast agents provide additional information in the hepatobiliary phase; specifically, washout or hypointensity of a suspicious lesion during this phase favors malignancy [35]. Furthermore, combined evaluation with contrast sequences and DWI appears to increase the detection of nodules [36] but has a limited role in the characterization of nodules.

*Practical points: MRI is the preferred next cross-sectional imaging tool for characterizing suspicious liver nodules detected on US. Hepatobiliary contrast products, where available, can be used off-label to increase the diagnostic accuracy. Further, in centers where MRI is used as a screening tool, if no nodules are visible on the pre-contrast sequences, avoiding contrast injection should be considered to decrease the risk of gadolinium brain deposition.*

### Computed tomography

Magnetic resonance imaging, when available, is usually preferred over CT for liver tissue characterization in children. Contrast administration is mandatory in CT liver diagnosis. When using CT, ionizing radiation dose reduction strategies must be adopted; importantly, to limit radiation exposure the pre-contrast phase should not be performed [37]. Sedation might not be necessary with newer CT scanners, which have very fast acquisition times. Because characterization of liver nodules on CT is based on their vascularity, possible contrast washout in benign nodules remains a pitfall to diagnosis.

*Practical points: CT should be used only when MRI is contraindicated/not available. Dedicated radiation dose reduction protocols are needed to reduce irradiation.*

### Elastography

There are several noninvasive methods to evaluate liver stiffness. Shear-wave elastography uses shear waves to quantify tissue elasticity [38]. In adults, the examination must be performed following strict rules: 2–4 h of fasting before the procedure, careful patient and probe positioning, and appropriate sampling and placement of the region of interest [39]. In children, because of the small size of cohorts, the cut-off values are not validated; also, performing the exam is more challenging because fasting, cooperation and breath-holding are not possible in younger age groups.

There is no recommendation on the use of US-based elastography in children with Fontan circulation. Although there appears to be a correlation between the degree of fibrosis and elastography values in chronic liver diseases, these might differ based on pathology [40].

In children with Fontan circulation it has been shown that liver stiffness correlates with the degree of fibrosis and hepatic afterload [41]. However, one should bear in mind that a single sampling does not reflect the entirety of the organ, that values can vary from different manufacturers, and that increased values can also be caused by hepatic congestion [42–44]. On the other hand, because shear-wave elastography US methods allow for sampling different parts of the liver, this method might help to obtain a more accurate evaluation of the heterogeneity of liver stiffness; the use of the median value might be closer to the “global stiffness” of the organ, so it might be considered of practical interest for longitudinal follow-up in individual Fontan patients.

MR-based elastography employs a mechanical driver placed on the right upper abdominal quadrant that transmits shear waves whose speed is slower in softer tissues and faster in stiffer ones. A two-dimensional (2-D) gradient recalled echo sequence is used at 1.5-tesla (T), whereas a 2-D gradient recalled echo or a 2-D spin-echo echoplanar sequence is used at 3 T [45]. The advantage over US-based elastography is the larger volume of liver studied; however, MR elastography is more complex to perform. Both techniques are biased by other possible conditions, such as inflammation, venous congestion and fat.

Few studies with histology and MR elastography values are available. However, when biopsy is performed, liver stiffness was reported to correlate, in an adult cohort, with fibrosis score as well as with time since operation, mean Fontan pressure, Model for End-Stage Liver Disease score, gamma-glutamyltransferase and creatinine levels, and pulmonary vascular resistance index; HCC also showed increased stiffness in one study [46, 47].

Other MR techniques that might be used to evaluate liver fibrosis are T1, T2 and T1ρ mapping [48–50]. Normal reference values have not been validated in children and local reference values on individual scanners need to be established. T1 and T2 mapping might also be influenced by inflammation, edema and iron overload, although a correction factor for the last exists for T1 mapping. T1ρ mapping appears to be more directly related to fibrosis alone [45]. However, little has been published on the subject.

*Practical points: When doing US, it is advisable to perform US elastography to assess global liver stiffness and to compare these data to previous examinations of the same child performed on the same US machine. Different US manufacturers might show different values. Further, elastography values are influenced by liver fibrosis but also by venous congestion/inflammation, so the interpretation must be considered in a clinical perspective. MR elastography can be added to the MR protocol, when available and when this is performed for other reasons (liver or cardiac imaging); these values can also be influenced by inflammation, congestion and fat.*

## Liver biopsy

Literature on the role of liver biopsy, performed via a transjugular or percutaneous approach, to assess liver fibrosis and cirrhosis and its correlation with other findings is sparse and sometimes contradictory. There are three possible scenarios: biopsy can be performed as (1) part of the follow-up, (2) before heart transplant or (3) to characterize liver nodules. In some centers, liver biopsy is included in the regular follow-up for Fontan patients, but most of the available data come from retrospective studies.

A modest positive correlation between liver fibrosis and time from surgery was reported in predominantly non-symptomatic adolescents and young adults, with no correlation to any hemodynamic risk factor, in a retrospective study including 67 individuals [51]. Complications following liver biopsy were reported as similar to those observed in non-Fontan patients [52]. In a prospective study of 38 adults undergoing liver biopsy as part of the Fontan follow-up, severe liver fibrosis was found in the majority of cases, without significant correlation with imaging or with symptoms [14]. In a prospective cohort of 17 adolescents, elastography correlated with time since Fontan operation but not with histopathology; the authors therefore suggested that elastography might be a useful tool to determine the evolution of liver stiffness over time in single patients [53] rather than being a substitute for histology.

In an adult cohort of 68 patients, either symptomatic or asymptomatic, retrospectively reviewed histology was abnormal in every case, with sinusoidal dilatation and sinusoidal fibrosis almost always present [54]. Interestingly, in a retrospective study of 49 adolescents and young adults, a significant correlation between histology, Fontan pressures, MR elastography and time since surgery was reported [55]. As mentioned, other studies have found a positive correlation between histology and MR elastography [46, 47].

Liver biopsy is otherwise usually performed before heart transplant, whether or not this is associated with liver transplant [56]. In this scenario consensus is not universal, especially about the capability of histology to predict whether the patient will survive heart-only transplant, or to influence the best timing for surgery [52].

Liver nodules are a separate issue. These occur frequently in Fontan patients and the imaging findings can be more difficult to interpret than in other types of chronic liver disease. In a prospective study of 155 adults, liver nodule prevalence was 47.7% on cross-sectional imaging. Eight hypervascular nodules showed contrast washout; HCC was diagnosed on biopsy on two of these [25]. Biopsy of suspicious liver nodules, as well as for assessing level of alpha-fetoprotein, appears therefore to be recommended.

*Practical points: The usefulness of liver biopsy as a screening tool in liver follow-up has not been demonstrated. Therefore, we do not recommend it. When HCC is suspected on imaging, biopsy of the nodule should be performed when possible*.

## Liver follow-up post Fontan circulation: consensus-based imaging algorithm

Follow-up varies largely among centers. The American Heart Association expert consensus [3] considers it reasonable to start surveillance during childhood and repeat it every 3–4 years; the association’s protocol includes liver US as an in-depth (non-basic) test, whereas liver CT or MRI, US or MR elastography, and biopsy are considered investigational tests. For children 12–18 years of age, the American Heart Association recommends performing surveillance every 1–3 years; biopsy is the investigational test, whereas liver US, liver CT or MRI, and US or MR elastography are all considered in-depth tests.

Greenway et al. [57] recommended noninvasive liver screening, including US, to be performed annually, and CT and MRI to be performed in cases of an abnormality found on US. In a review by Komatsu et al. [58], the authors suggested that considering the risk of HCC even at a young age in Fontan-associated liver disease, follow-up should commence at 7–8 years of age, with noninvasive tests such as US, CT and MRI indicated as suitable.

According to a recent publication by Dillman et al. [4], US should be avoided as unsuitable to assess manifestations of Fontan-associated liver disease such as portal hypertension and hepatic neoplasm; rather, the authors suggested that contrast-enhanced MRI and MR elastography be performed, beginning at age 13, every other year (with CT as an alternative if MRI is contraindicated, and US shear-wave elastography in the off years in this case) as the basic follow-up protocol. The authors also recommended that cross-sectional studies be performed more frequently in cases where imaging features raise a moderate suspicion for neoplasm, and that biopsy be done in cases of high suspicion for neoplasm or in the pre-transplant setting [4].

With the aim of addressing the current practice in European institutions, the ESPR Abdominal Task Force recently invited the members of ESPR to take part in a survey [5]. The results of the survey have been the basis for discussion among members of the task force, with the goal to establish consensus-based guidelines.

The area of major disagreement among survey respondents was the appropriate age for beginning liver follow-up, not surprisingly, because very little indication is available in the literature. However, as underlined by Komatsu et al. [58], malignant hepatic tumors have been described in individuals younger than 18 years and have shown a poorer prognosis than HCC secondary to other causes [16, 20–23]. Because of the cases described, it appears reasonable to propose a more aggressive follow-up, ideally starting in infancy after surgery, although this could be modified after more data and experience are gained. Moreover, because all Fontan patients have some degree of liver abnormality with increased risk of developing liver cirrhosis from their pre-existing hemodynamic condition, it would be unwise to wait for the appearance of symptoms.

Every center responding to our survey performed (at least) US as the basic screening tool, likely because of its numerous advantages, including wide availability and relatively low cost. US elastography could be easily added to this examination, although results must be interpreted cautiously given that fibrosis and congestion cannot be differentiated and that values vary among manufacturers and age range [59, 60].

Given the availability and repeatability of US and US elastography, we recommend an annual screening to detect progression of liver condition, starting in infancy after surgery.

Although studies are needed on the correlation among liver stiffness, portal hypertension and the occurrence of HCC, an annual surveillance should allow for detection of significant pathology and provide further data on Fontan-associated liver disease development. Although the role of CEUS is apparently limited to the characterization of liver nodules, this examination could be added in centers with adequate expertise when the expected benefit is superior to the theoretical risk, noting that CEUS is still off-label in Europe.

**Fig. 2:**
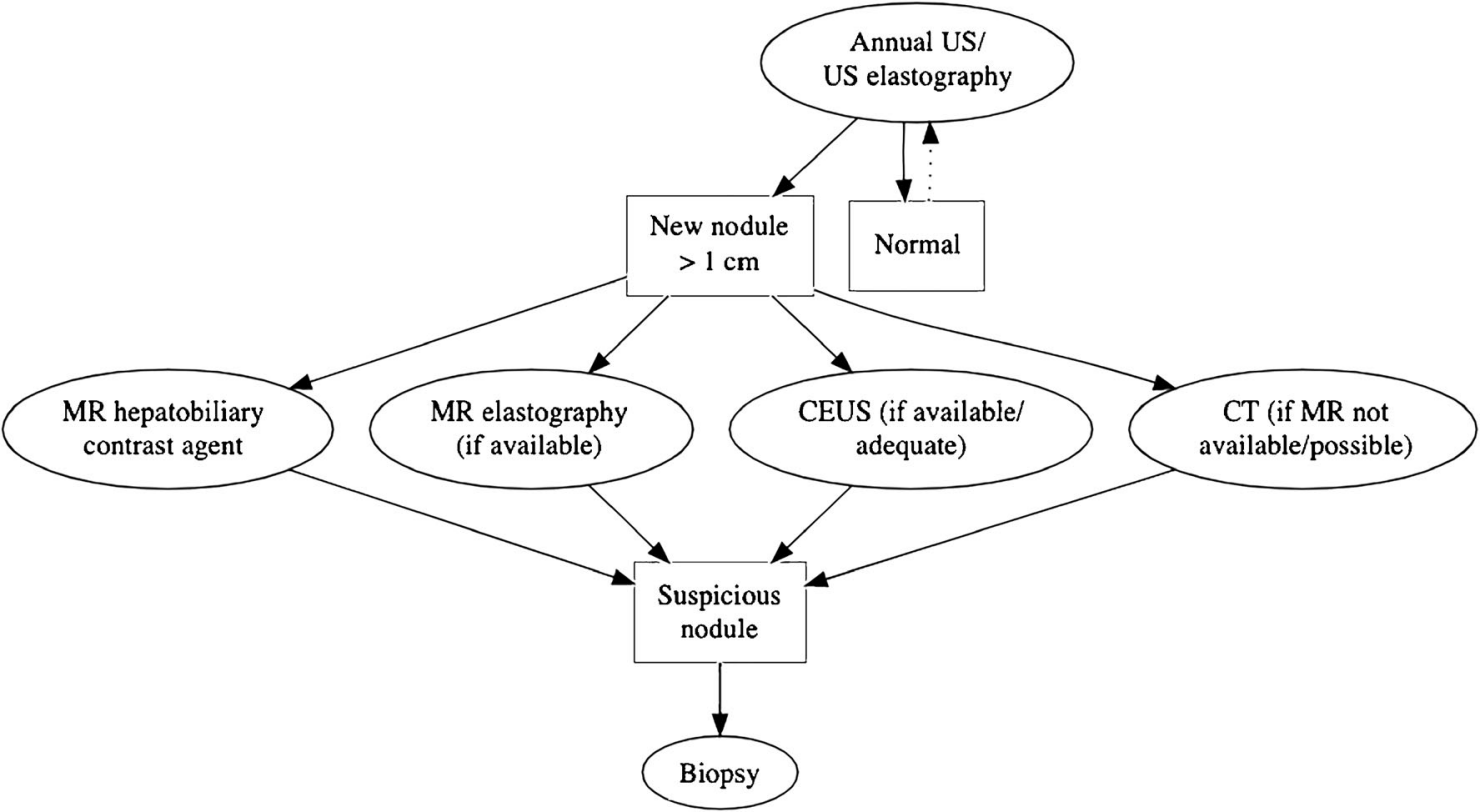
European Society of Paediatric Radiology (ESPR) Abdominal Task Force proposal for Fontan-associated liver disease surveillance. *CEUS* contrast-enhanced ultrasound

Additional cross-sectional imaging is unanimously considered the investigational tool in cases of new liver nodules on US; MRI is preferred over CT, and the use of hepatobiliary contrast agent offers some advantages in the diagnostic accuracy. In cases of suspected malignant nodules, biopsy is recommended when possible, alongside assessment for alpha-fetoprotein (Fig. 2).

## Conclusion

In this paper, we highlight the need for a consensus on imaging follow-up in Fontan patients. Although it is impossible to establish evidence-based guidelines at this time, there is wide agreement on the higher risk for Fontan patients of developing fibrosis/cirrhosis and HCC at a young age. Consequently, performing liver imaging follow-up is necessary for early detection of a possible malignancy. In addition, monitoring liver fibrosis development might be important as part of the cardiac evaluation of the optimal timepoint for a transplantation.

The proposal of the ESPR Abdominal Task Force is to adopt a homogeneous strategy that will grant comparability among centers. That is expected to facilitate prospective studies to clarify the role of each modality for the detection of different liver complications and then elaborate an appropriate imaging protocol for follow-up. Moreover, we recommend close multidisciplinary cooperation, especially with pediatric cardiologists, cardiac surgeons and hepatologists, considering the complexity of this condition that requires a multisystemic approach.
